# Increased visceral arterial tortuosity in Marfan syndrome

**DOI:** 10.1186/s13023-020-01369-w

**Published:** 2020-04-15

**Authors:** Bence Ágg, Bálint Szilveszter, Noémi Daradics, Kálmán Benke, Roland Stengl, Márton Kolossváry, Miklós Pólos, Tamás Radovits, Péter Ferdinandy, Béla Merkely, Pál Maurovich-Horvat, Zoltán Szabolcs

**Affiliations:** 1 Heart and Vascular Center, Semmelweis University, Városmajor u. 68, Budapest, H-1122 Hungary; 2 Hungarian Marfan Foundation, Városmajor u. 68, Budapest, H-1122 Hungary; 3 Department of Pharmacology and Pharmacotherapy, Semmelweis University, Üllői út 26, Budapest, H-1085 Hungary; 4 MTA-SE Cardiovascular Imaging Research Group, Heart and Vascular Center, Semmelweis University, Városmajor u. 68, Budapest, H-1122 Hungary

**Keywords:** Marfan syndrome, Arterial tortuosity, Visceral arteries, Risk stratification

## Abstract

**Background:**

Clinical evidence suggests that the currently recommended approach to estimate the risk of aortic dissection in Marfan syndrome (MFS) is not reliable enough. Therefore, we investigated the possible role of visceral arterial tortuosity in the risk stratification.

**Methods and results:**

Splenic and renal arteries of 37 MFS patients and 74 age and gender matched control subjects were segmented using CT angiography imaging. To measure tortuosity, distance metric (DM), sum of angles metric (SOAM), inflection count metric (ICM), and the ratio of ICM and SOAM (ICM/SOAM) were calculated. DM of the splenic, right and left renal artery was significantly higher in MFS patients than in controls (2.44 [1.92-2.80] vs. 1.75 [1.57-2.18] *p* < 0.001; 1.16 [1.10-1.28] vs. 1.11 [1.07-1.15] *p* = 0.011; 1.40 [1.29-1.70] vs. 1.13 [1.09-1.23] *p* < 0.001, respectively). A similar tendency for ICM and an opposite tendency for SOAM were observed. ICM/SOAM was significantly higher in the MFS group compared to controls in case of all three arteries (73.35 [62.26-93.63] vs. 50.91 [43.19-65.62] *p <* 0.001; 26.52 [20.69-30.24] vs. 19.95 [16.47-22.95] *p* < 0.001; 22.81 [18.64-30.96] vs. 18.38 [15.29-21.46] *p* < 0.001, respectively). MFS patients who underwent aortic root replacement had increased right and left renal DM and ICM/SOAM compared to MFS patients without surgery.

**Conclusion:**

To our knowledge this is the first demonstration of increased arterial tortuosity in MFS on visceral arteries. Visceral arterial tortuosity, dominated by curves of lower frequency but higher amplitude according to the observed opposite tendency between the DM and SOAM metrics, could be a possible new predictor of serious manifestations of MFS.

## Introduction

Marfan syndrome (MFS) is a genetically determined, autosomal dominantly inherited connective tissue disorder that involves the skeletal, the ocular and the cardiovascular systems. The incidence of the syndrome is about 2-3 per 10,000 individuals [1]. MFS is caused by a wide variety of mutations in the fibrillin-1 (*FBN1*) gene [2, 3]. Besides the obvious structural role of FBN1 in the extracellular matrix, additional effects of the mutation can be explained by its role in the regulation of the availability of transforming growth factor-β (TGF-β) [4, 5].

The most severe, life-threatening manifestation of MFS is aortic dissection that necessitates acute surgical intervention. It is of utmost importance to choose the optimal timing of properly planned operative solution, as if an acute intervention was needed, more reoperations could follow it [6]. Moreover, we observed that late overall death strongly correlates with acutely performed operations [7]. However, prophylactic aortic root replacement executed at an unreasonably young age entails more reoperations, and also lifelong anticoagulation in case of mechanical valve implantations [8]. Aortic diameter and family history are most frequently evaluated as predictors of aortic dissection in the current surgical criteria, however observations in the clinical practice suggest that these parameters on their own are not reliable enough [9–11]. Therefore, extending the current criteria and establishing a clinically relevant score system is indispensable to be able to precisely predict the risk and the expected onset of aortic dissection.

Previous findings analyzing arterial tortuosity of MFS patients has shown that assessing this parameter could improve cardiovascular risk stratification [12, 13]. These studies recognized that tortuosity of the vertebral arteries and the aorta is increased in MFS compared to the normal population. Moreover, within the MFS population the level of tortuosity correlated with the severity of aortic involvement [12, 13]. However, as skeletal deformities characteristic of the syndrome could obviously influence the geometry of the studied arterial segments, special care should be taken when assessing these metrics in patients with severe skeletal deformities. For instance scoliosis, a very frequent skeletal feature of MFS, due to the close proximity of the vertebral arteries and the aorta to the spine, can have a considerable influence on tortuosity, while among others, pectus excavatum can easily dislocate the aorta, and therefore the tortuosity of the vessels could be altered by these deformities.

To identify possible predictors of serious aortic involvement, that are not dependent on the effect of skeletal deformities of MFS, here we investigated for the first time the tortuosity of the visceral arteries, namely the splenic, the right and left renal arteries. Furthermore, to get a full picture of tortuosity in MFS we also calculated tortuosity metrics that has not been studied in MFS so far achieving a precise geometric description of the investigated vessels.

## Methods

### Patient selection

To avoid exposure of patients to additional X-ray radiation our study was implemented in a retrospective fashion utilizing already available imaging data. For our analysis we included all MFS patients with available helical CT angiography (CTA) images (*n* = 114) from the Hungarian Marfan Registry [14]. After the assessment of coverage of the analyzed visceral arteries by the CT slices and the exclusion criteria, CT images of 37 MFS patients were kept for further analysis. In 4 cases image noise impeded the analysis of the target vessels, also in 67 cases the coverage of the investigated arteries was not appropriate or venous phase was only available due to the given contrast protocol. We excluded 6 patients in whom visceral arterial tortuosity could not be measured due to the presence of any pathological structure that could influence measurements by possibly altering the geometry of these arteries. Most common pathologies included intra-abdominal tumors (renal, pancreatic or liver malignancies), pancreas pseudocysts, large renal cysts and aortic aneurysms.

According to the current recommendations, in case of all 37 selected patients the diagnosis of MFS was reevaluated and confirmed by assessing the clinical criteria of the revised Ghent nosology [15]. In addition to the established clinical diagnosis, in 16 cases pathogenic or likely pathogenic mutation was already identified in the FBN1 gene within our ongoing genetic testing program. Patient characteristics of the MFS group including the prevalence of risk factors for atherosclerosis are summarized in Table 1.

**Table 1:** Patient characteristics of the severity groups of Marfan syndrome patients including the presence of risk factors for atherosclerosis

Groups	A (*n* = 5)	B (*n* = 12)	C (*n* = 20)	All (*n* = 37)
Age at CT (years)	32.4 ± 2.6	37.5 ± 6.6	42.3 ± 14.3	39.4 ± 11.6
Male	2	8	12	22
Involvement of cardiovascular system	No intervention required	Mild involvement required intervention	Severe cardiovascular involvement	Involvement of varying degree
Anthropometric (measured)				
Height (cm)	184.0 [180.0-185.0]	194.0 [182.0-199.2]	187.5 [181.2-195.8]	186.0 [181.5-197.5]
Arm span (cm)	183.0 [182.0-187.0]	191.0 [189.0-210.0]	186.0 [184.2-204.5]	189.0 [183.0-204.0]
Lower segment (cm)	90.0 [90.0-99.0]	95.0 [94.0-104.0]	98.0 [93.2-99.8]	97.0 [91.8-103.0]
Foot size (cm)	28.0 [25.3-28.0]	29.0 [26.7-30.2]	27.7 [26.7-29.7]	28.0 [26.7-30.0]
Weight (kg)	70.0 [60.0-72.0]	74.0 [63.8-93.5]	82.0 [72.8-96.5]	75.0 [65.0-93.0]
Anthropometric (calculated)				
Arm span to height ratio	1.01 [1.01-1.02]	1.02 [1.00-1.04]	1.02 [1.01-1.07]	1.02 [1.01-1.05]
Upper segment to lower segment ratio	1.00 [0.82-1.06]	0.92 [0.84-0.96]	0.90 [0.86-0.97]	0.92 [0.85-1.01]
Body Mass Index (BMI; kg/m^2^)	20.5 [18.5-21.3]	20.6 [19.6-24.1]	23.0 [21.4-26.0]	21.5 [19.6-25.2]
Body surface area (m^2^) - Mosteller	1.90 [1.73-1.92]	2.01 [1.81-2.27]	2.08 [1.92-2.26]	1.97 [1.81-2.26]
Ghent nosology				
Positive family history (%)	80.0%	58.3%	35.0%	48.6%
Systemic score (SSc)^*^	5.0 [2.0-7.0]	8.0 [6.5-9.0]	8.0 [5.8-9.0]	8.0 [5.0-9.0]
SSc < 7 pts. (%)	60.0%	25.0%	30.0%	32.4%
SSc 7-10 pts. (%)	40.0%	58.3%	65.0%	59.5%
SSc > 10 pts. (%)	0.0%	16.7%	5.0%	8.1%
Risk factors for atherosclerosis				
Hypertension (%)	0.0	66.7	25.0	35.1
Hyperlipidemia (%)	0.0	8.3	15.0	10.8
Smoking (%)	0.0	25.0	5.0	10.8
Diabetes (%)	0.0	0.0	5.0	2.7
History of coronary artery disease (%)	0.0	0.0	5.0	2.7

*SSc Systemic score

Control subjects were selected from our clinical imaging database and were matched for age and sex with a control-to-case ratio of 2:1, thus a total of 74 control CTA sequences were analyzed. Only patients without the diagnosis of MFS or any related connective tissue disorder like vascular Ehlers-Danlos syndrome, Loeys-Dietz syndrome or Shprintzen-Goldberg syndrome were included in the control group. Furthermore, the presence of possibly distorting pathological structures or imaging artifacts, as described in case of the MFS group, were also applied as exclusion criteria for control patients. Basic characteristics of the control individuals and the prevalence of risk factors for atherosclerosis are shown in Table 2.

**Table 2:** Patient characteristics of the control group including the presence of risk factors for atherosclerosis

	Control group (*n* = 74)
Age at CT (years)	39.7 ± 11.5
Male	44
Risk factors for atherosclerosis	
Hypertension (%)	32.4
Hyperlipidemia (%)	6.8
Smoking (%)	18.9
Diabetes (%)	5.4
History of coronary artery disease (%)	2.7

### Severity groups

Patients with MFS were classified into three groups based on the severity of aortic involvement, in a similar manner as in a previous study [16]:

*Group A (n = 5)* – Patients without aortic involvement requiring surgery at the time of the CT scan:

- no aortic dissection
- no significant aortic valve insufficiency
- diameter of the ascending aorta < 45 mm

*Group B (n = 12)* – Patients who had undergone elective surgery of the ascending aorta before the CT scan because of mild aortic manifestations of the syndrome according to the following surgical indication criteria:

- diameter of the ascending aorta between 45 and 50 mm OR diameter at the level of the sinus of Valsalva between 45 and 48 mm with grade I-II aortic regurgitation
- AND aortic dilation rate of > 2 mm/year OR aortic dissection in the family history

*Group C (n = 20)* – Patients who had been operated for either annuloartic ectasia or type A aortic dissection before the CT scan according to the following indication criteria:

- *Annuloaortic ectasia:* diameter of the ascending aorta > 50 mm OR diameter at the level of the sinus of Valsalva > 48 mm with grade III-IV aortic regurgitation
- *Aortic dissection:* type A aortic dissection confirmed by CT

The above surgical indication criteria were determined according to the ESC/EACTS guidelines [10, 11]. Patient characteristics of the severity groups are available in Table 1.

### CT angiography

**Fig. 1:**
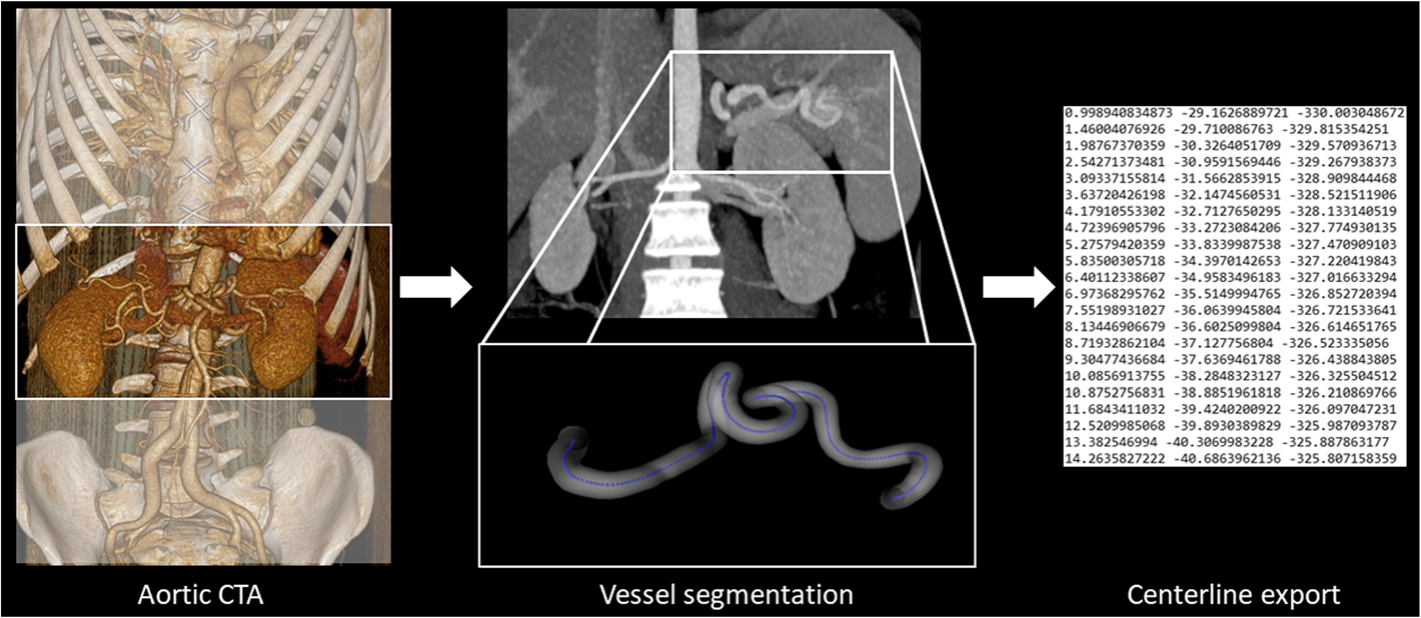
To measure tortuosity abdominal CT angiography images of patients with MFS and age and gender matched controls were selected. After manual segmentation of the vessels 3D coordinates of the centerlines were exported to calculate tortuosity metrics

We evaluated contrast enhanced CT images of the abdominal aorta in all patients (Fig. 1). Firstly, we identified all MFS patients who underwent 256-slice CT angiography (Philips Brilliance iCT) at our Institute and assessed arterial phase images for tortuosity measurements. Control subjects with aortic CT angiography of the abdomen were selected based on our clinical imaging database. Aortic CTA images were analyzed with a slice thickness of 1-2.5 mm, whereas the amount of contrast agent and acquisition settings varied based on local protocols. Images were reconstructed using traditional Filtered Back Projection or Hybrid-type iterative reconstruction.

### Image segmentation and centerline export

In order to generate centerlines for further calculations, we loaded the selected CT datasets to a dedicated workstation. Images were analyzed using Medis QAngio CT (v.3.1.0.1) by a single reader, who was blinded to patient’s clinical characteristics. Images were evaluated in a random fashion. The reader manually segmented the splenic and both renal arteries placing markers in the vessel lumen. Additional adjustment of the centerlines was carried out, if necessary. The centerline of the splenic artery was extracted from the coeliac trunk to the bifurcation at the hilus of the spleen. The renal artery on both side was identified from its aortic origin to the renal hilus, selecting the largest branch with a vessel diameter of more than 1.5 mm. Thereafter, we saved the segmented arteries and exported all centerlines using the Medis QAngio CT 3D Workbench (v 0.8) in a text format containing the 3 dimensional vessel coordinates.

### Tortuosity metrics

**Fig. 2:**
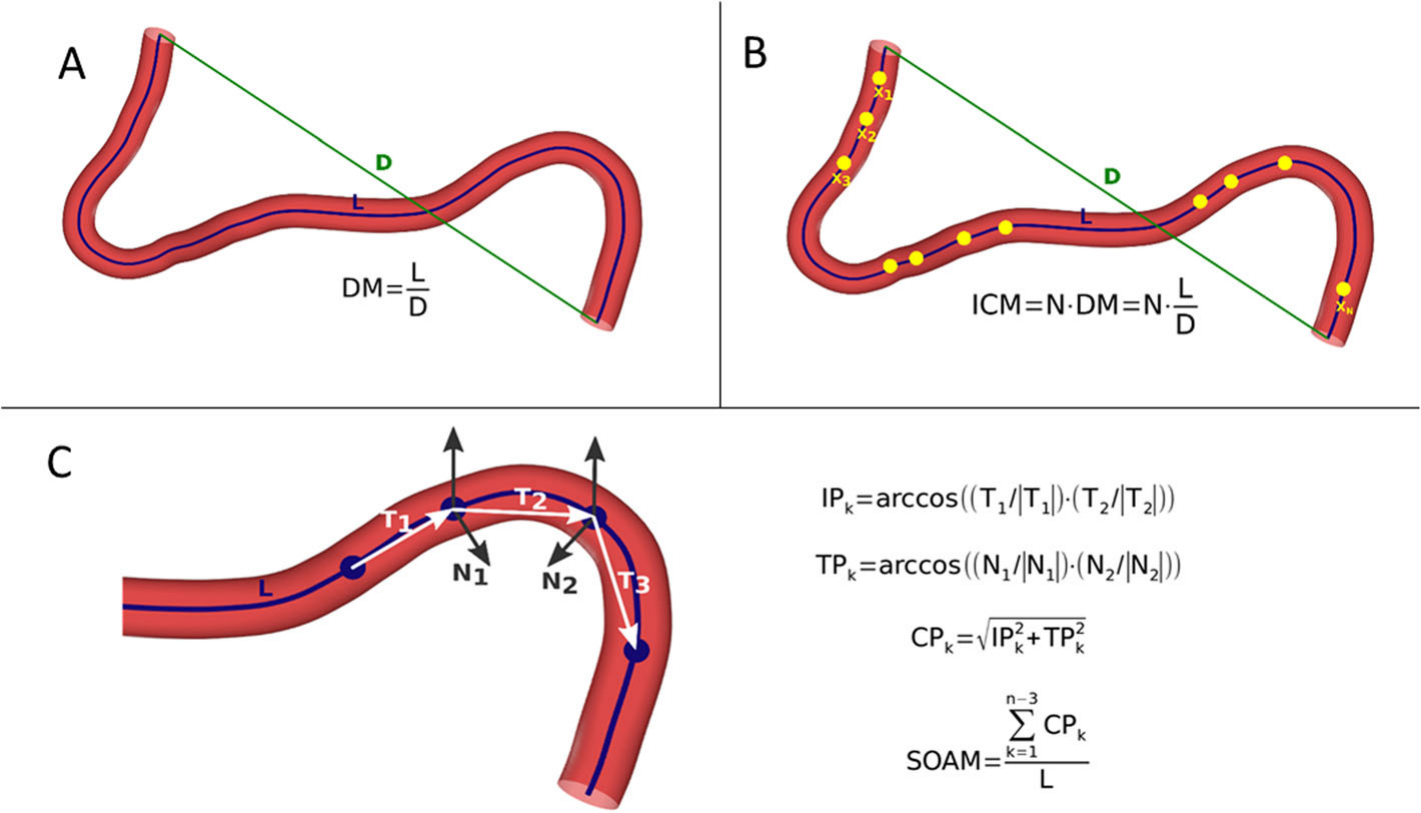
Schematic illustration of the algorithms used to calculate tortuosity metrics as described by Bullitt et al. Distance Metric (DM) provides a ratio between the actual path length of a meandering curve (L, blue centerline) and the linear distance (D, green straight line segment) between the endpoints **(a)**. Inflection count metric (ICM) is calculated by counting the inflection points, so those points where the curve changes from convex to concave (highlighted as yellow dots in the picture). The number of inflection points (N) is normalized by multiplying it by the DM **(b)**. Sum of angles metric (SOAM) is assessed by subdividing the arterial centerline into small segments (T_1-3_, white arrows) and summing the in-plane (IP_k_) and torsional angles (TP_k_) between these segments **(c)**

To assess the geometric properties of the analyzed arteries three tortuosity metrics were calculated for each vessels namely distance metric (DM), three-dimensional (3D) version of the inflection count metric (ICM) and the 3D version of the sum of angles metric (SOAM) as illustrated in Fig. 2. The algorithms to calculate these three tortuosity metrics were implemented as described by Bullitt et al. [17] in JavaScript (Node.js) language as a server-side software plugin for the electronic version of the Hungarian Marfan Registry. Implementation for all three metrics were validated against the synthetic datasets published along with the original definition of the algorithms [17]. To assess the relative contribution of amplitude and frequency to tortuosity values, indicated by ICM and SOAM, respectively [17], in addition to the three metrics described by Bullit et al. a fourth derived metric was also calculated for each investigated arterial segment as the ratio between ICM and SOAM (ICM/SOAM).

### Statistical analysis

Statistical analysis of the collected data was performed with the R software environment [18]. Association between two classifications (risk factors for atherosclerosis in the MFS and the control group) was examined by Fisher’s exact test. Normality of the datasets was assessed by the Shapiro-Wilk test. As tortuosity metrics in our study did not follow normal distribution, tortuosity values are reported as medians with interquartile ranges (IQR) and further analysis was performed using non-parametric tests. Accordingly, for comparing two groups Mann-Whitney U-test was applied. Kruskal-Wallis-test was used to compare multiple groups and pairwise Mann-Whitney U-test with Benjamini-Hochberg adjustment for multiple comparisons was performed as post-hoc test. Spearman’s rank correlation coefficient was calculated to assess the relation between tortuosity metrics and other variables.

## Results

### Visceral arterial tortuosity in Marfan syndrome compared to controls

Tortuosity metrics of the three investigated visceral vessels followed a normal distribution neither in the MFS group nor in control individuals.

**Fig. 3:**
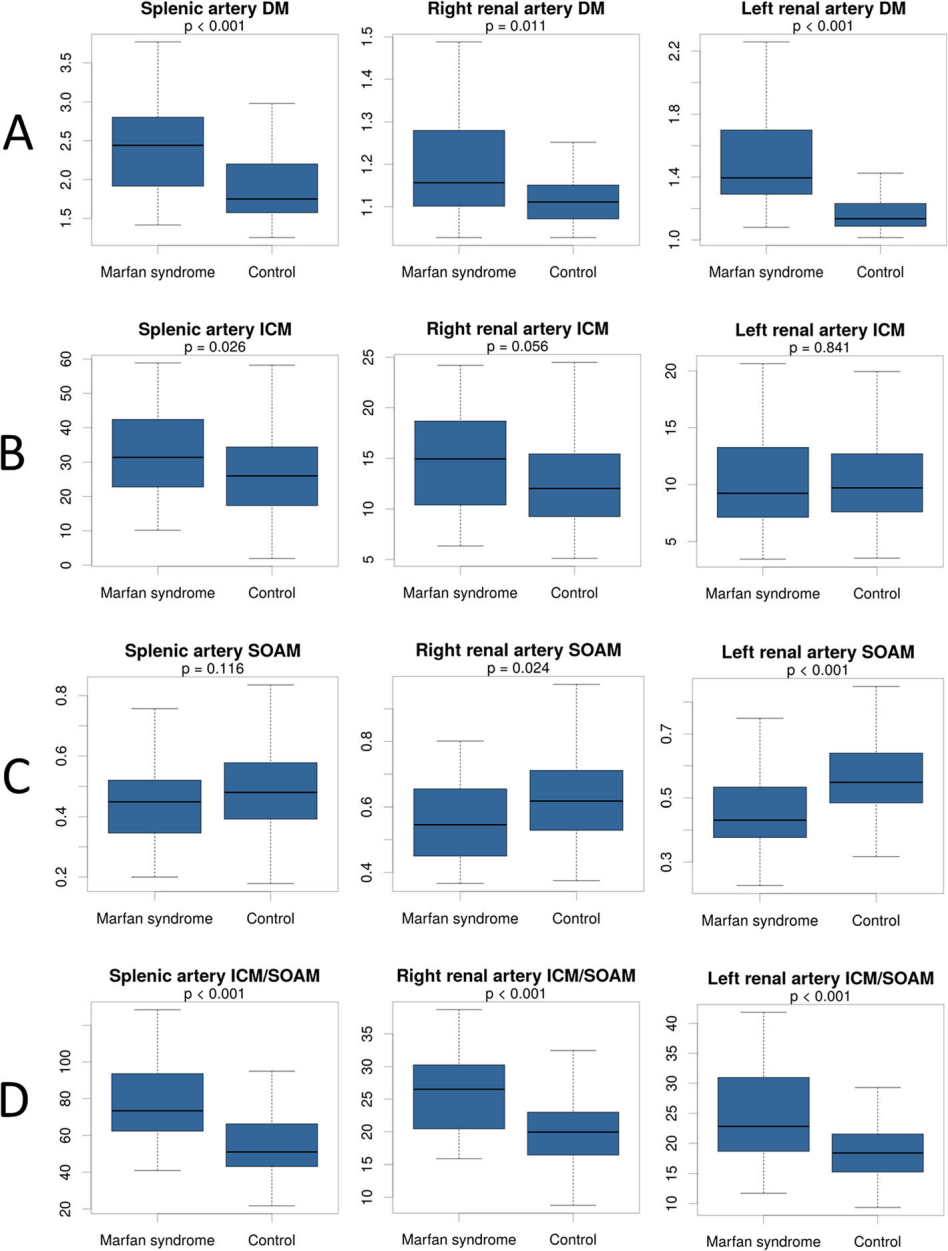
Tortuosity of the splenic, right and left renal arteries in MFS patients compared to controls. DM was significantly increased in case of all three vascular segments in MFS compared to controls (**a**). ICM followed a similar tendency (**b**) SOAM changed in the opposite direction **(c)**, while the derived ICM/SOAM metric was significantly higher in the MFS cases on all three vessels (**d**)

In MFS patients DM was significantly increased compared to control individuals in case of the splenic and the right and left renal arteries (2.44 [1.92-2.80] vs. 1.75 [1.57-2.18] *p* < 0.001; 1.16 [1.10-1.28] vs. 1.11 [1.07-1.15] *p* = 0.011; 1.40 [1.29-1.70] vs. 1.13 [1.09-1.23] *p* < 0.001 respectively) (Fig. 3a).

ICM showed similar changes in case of the splenic and right renal arteries (31.43 [22.75-42.39] vs. 26.02 [17.86-34.30] *p* = 0.026; 14.95 [10.65-18.53] vs. 12.03 [9.26-15.17] *p* = 0.056 respectively), however the difference was significant only in case of the splenic artery. No significant difference in ICM could have been observed in case of the left renal artery (9.26 [7.13-13.27] vs. 9.73 [7.63-12.72] *p* = 0.841) (Fig. 3b).

SOAM was significantly lower in the MFS group than in the control individuals in case of the right and left renal arteries (0.55 [0.45-0.65] vs. 0.62 [0.53-0.71] *p* = 0.024; 0.43 [0.38-0.53] vs. 0.55 [0.49-0.64] *p* < 0.001 respectively). The tendency was the same in case of the SOAM of the splenic artery however the *p*-value did not reach the significance level (0.45 [0.35-0.52] vs. 0.48 [0.39-0.58] *p* = 0.116) (Fig. 3c).

The derived ICM/SOAM was significantly increased in the MFS group compared to controls in case of all three arteries, namely the splenic, the right and left renal arteries (73.35 [62.26-93.63] vs. 50.91 [43.19-65.62] *p* < 0.001; 26.52 [20.69-30.24] vs. 19.95 [16.47-22.95] *p* < 0.001; 22.81 [18.64-30.96] vs. 18.38 [15.29-21.46] *p* < 0.001) (Fig. 3d).

When comparing the MFS and control groups there was no significant difference in the prevalence of risk factors for atherosclerosis including hypertension (*p* = 0.832), hyperlipidemia (*p* = 0.478), smoking (*p* = 0.413), diabetes (*p* = 0.663) and history of coronary artery disease (*p* = 1.000).

### Visceral arterial tortuosity in the severity groups of Marfan syndrome patients

**Fig. 4:**
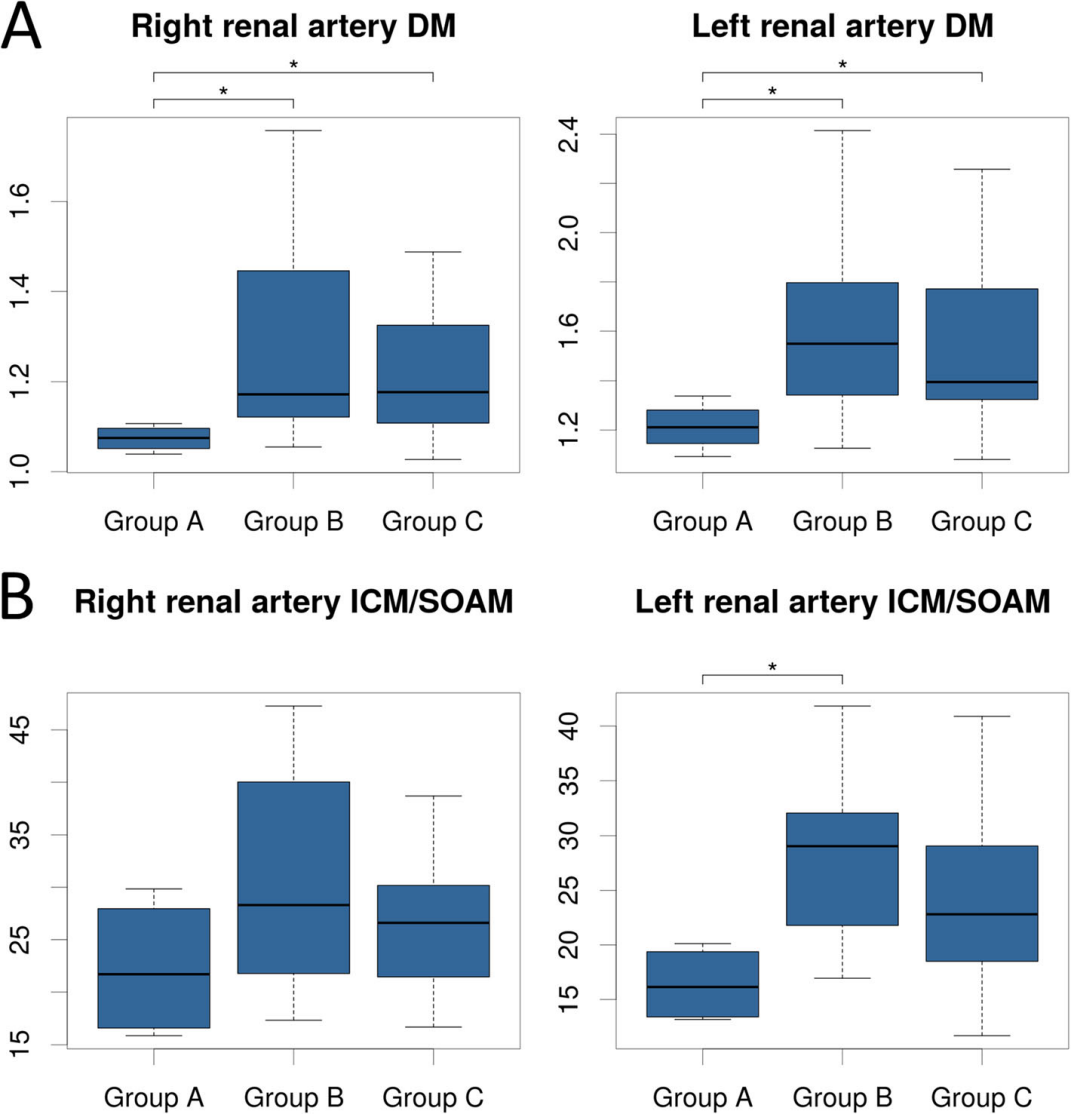
Right and left renal arterial tortuosity in the severity groups of MFS patients. Group A - patients without aortic involvement. Group B - patients who had undergone elective surgery of the ascending aorta. Group C - patients who had been operated for either annuloaortic ectasia or type A aortic dissection. Both DM **(a)** and ICM/SOAM **(b)** of the two renal arteries were increased in patients who underwent surgery (Group B and Group C) compared to the non-operated group (Group A)

In case of right and left renal DM a significant difference was observed between the severity groups of MFS patients (Kruskal-Wallis *p* = 0.045 and 0.049, respectively) (Fig. 4a). Particularly, when compared to patients who underwent ascending aortic surgery because of various indications (Group B, Group C), patients without significant aortic involvement (Group A) had significantly lower right (*p* = 0.039 and *p* = 0.039) and left renal DM (*p* = 0.041, *p* = 0.041, respectively). ICM and ICM/SOAM followed a similar tendency, however the difference was significant only when comparing the left renal ICM/SOAM of Group A and Group B (Kruskal-Wallis *p* = 0.040; *p* = 0.023) (Fig. 4b).

## Discussion

In this study we investigated the geometrical features of visceral arteries in MFS patients by analyzing abdominal CT angiography images and calculating multiple advanced measures of arterial tortuosity. With this approach we found increased tortuosity on the splenic, left and right renal arteries of MFS patients compared to control individuals. We also identified differences in tortuosity metrics in case of the severity groups of the studied MFS population indicating a possible role of visceral arterial tortuosity in the prediction of severe aortic manifestations of MFS.

It is well known that there is a whole spectrum of connective tissue disorders presenting with increased arterial tortuosity, including arterial tortuosity syndrome caused by the mutation of SLC2A10 gene [19], Loeys-Dietz syndrome with different mutations in genes encoding proteins involved in the TGF-β signaling (TGFB2, TGFB3, TGFBR1, TGFBR2, SMAD2 or SMAD3) [20] and autosomal recessive cutis laxa type IB caused by the mutation of FBLN4 gene [21].

After some incidental case reports of vascular tortuosity in MFS [22–24], in 2011 Morris et al. comprehensively investigated the possible role of vertebral tortuosity index (VTI) in the risk stratification of aortic involvement of MFS and other connective tissue disorders, and demonstrated its usefulness in predicting severe outcomes of these disorders [12]. Similarly increased aortic tortuosity index (ATI) was found to be a prognostic factor of increased rate of aortic dilation and type B aortic dissection in MFS [13].

While we have no doubt that VTI and ATI are valuable predictors of serious aortic involvement in MFS, we assume that these metrics should be evaluated carefully in cases with severe skeletal deformities, which are common manifestations of MFS. Both the aorta and the vertebral arteries are in close anatomical proximity to the spine, thus spinal deformities, including scoliosis which is present in at least half of the MFS population [25], may have a considerable effect on the value of both VTI and ATI. In two thirds of the patients with MFS varying degree of pectus excavatum is present, that could cause the displacement and compression of the heart especially in severe cases [26–28]. It is obvious that similarly to the heart, the ascending and thoracic segment of the descending aorta could be dislocated by a severe case of pectus excavatum, which could also modify the value of ATI. As visceral arteries are anatomically separated from those skeletal structures that exhibit deformities in MFS, we consider tortuosity metrics calculated for splenic and renal arteries mostly independent from the skeletal manifestations of MFS.

Nevertheless, similarly to skeletal manifestations of the syndrome, tortuosity of the abdominal aorta [13] could have an effect on the geometry of the visceral arteries. Although because of the retrospective design CT scans spanning the whole aorta was not available for each patient in this study, increased aortic tortuosity could be a possible explanation for the moderate differences in the trends observed in case of the tortuosity (especially ICM) values of the right and left renal arteries.

In the two above studies [12, 13] to measure tortuosity researchers used only VTI and ATI values that are calculated by dividing the length of the centerline and the distance between the two endpoints of the investigated vessel. However, this metric, which we called distance metric (DM) in our study according to Bullitt et al. [17], could be increased either if the vascular centerline consists of a single high amplitude curve, or if multiple lower amplitude but higher frequency features (e.g. sinusoidal curves) are present along the centerline of the vessel. Therefore, to differentiate between these cases further geometrical measures are necessary. To this end here we also calculated the three dimensional version of the inflection count metric (ICM), which is mostly sensitive to higher amplitude structures, and the sum of angles metric (SOAM), which tends to be increased in the presence of high frequency structures as described by Bullitt et al. [17]. According to our results, increased tortuosity of the splenic artery and renal arteries – indicated by significantly and consistently increased DM values – are accompanied by a tendency of increased ICM and decreased SOAM values, suggesting that geometry of these visceral arteries is dominated by higher amplitude and lower frequency curves in MFS patients compared to controls. This result is also supported by the significantly elevated ICM/SOAM values in case of the visceral arteries of MFS patients.

There is evidence that atherosclerosis could contribute to the development of increased arterial tortuosity [29, 30]. However, as we could not find any significant differences in the prevalence of the major risk factors for atherosclerosis between the MFS and the control group, we can likely exclude the effect of atherosclerosis as an explanation for our above findings.

A clear tendency of increased DM and ICM/SOAM of the right and left renal artery in patients who underwent aortic surgery (Group B, Group C) compared to patient who did not have surgery (Group A) indicates the possible predictive role of these tortuosity metrics in differentiating between the more benign and the more malignant form of MFS as proposed in our previous study for the classification of aortic involvement in this syndrome [31].

In our previous studies we demonstrated that increased serum TGF-β levels, the upregulation of MMP-3 in the peripheral blood mononuclear cells, the presence of striae atrophicae (stretch marks), alterations in the homocysteine metabolism and polymorphisms of the enzymes involved in this latter process could be related to more serious forms of aortic involvement in MFS [16, 31]. However, further investigation is necessary to elucidate whether the above findings together with the various tortuosity metrics could eventually enable us to develop a multivariate model and a score system to predict the severity of the aortic outcome.

Although the exact mechanism in the background of vascular tortuosity in MFS and related disorders is not clear, there are multiple hypotheses that could explain this geometrical alteration, including the pathological lengthening of the arteries [32], the potential role of increased TGF-β signaling [13] or the oxidative stress in case of arterial tortuosity syndrome [33]. Independently from the underlying mechanism, based on our current results and the increasing number of studies demonstrating arterial tortuosity of various arterial segments in MFS [12, 13], we propose that arterial tortuosity should be considered as a potential diagnostic criterion for MFS in addition to the currently used Ghent criteria [15].

### Limitations

We acknowledge the limitations of our study. Despite the relatively small population size, we were able to detect statistically significant differences in tortuosity values both in the case-control comparison and in the analysis of severity groups, which underscores the robustness of the applied tortuosity metrics. Furthermore, our study population is comparable in size to previous human MFS studies [34, 35].

Currently, prospective data on the clinical outcomes based on increased visceral arterial tortuosity are lacking, however we were able to detect significant differences in DM and the derived ICM/SOAM metric between MFS severity groups retrospectively.

The diagnosis of MFS based on phenotypic manifestations was unequivocal according to the revised Ghent nosology, and in 16 cases the causative mutation in FBN1 was already identified within our ongoing genetic testing program. Nevertheless, we could not entirely exclude the possibility that in a few cases out of the 21 patients without already established genetic diagnosis other disease like vascular Ehlers-Danlos syndrome, Loeys-Dietz syndrome or Shprintzen-Goldberg syndrome is present instead of MFS. However, based on the relative rarity of these genetic disorders compared to MFS, it most likely does not invalidate our findings [36–38].

Measurements were carried out by a single reader who was blinded to patients’ anamnestic and anthropometrical data to avoid potential bias. Due to unique variance in vessel length between MFS patients and controls we created centerlines with different 3D expansion, however the metrics used in this study are normalized for the distance between the two endpoints (DM, ICM) or the full length of the investigated arterial segment (SOAM). Therefore, differences in the length of the studied arterial segments should not influence our results. We matched subjects based on age and gender, even though, there might be additional factors that could influence patients’ vessel tortuosity.

## Conclusions and perspective

We demonstrated for the first time that tortuosity of visceral vessels, namely the splenic and the two renal arteries, is increased in MFS when compared to the non-MFS population. By using improved tortuosity metrics, we also showed that increased visceral arterial tortuosity is explained most likely by higher amplitude but lower frequency geometrical structures along the vessel centerlines. Also, tortuosity of visceral arteries has a low probability to be influenced by pectoral or spinal deformities characteristic to MFS, thus visceral arterial tortuosity can be a reliable parameter even in case of individuals presenting with severe skeletal manifestations of MFS. Based on the recent findings in the research of arterial tortuosity in MFS, we also propose, that tortuosity should be considered as a potential diagnostic criterion of MFS.

## Data Availability

The datasets used and/or analysed during the current study are available from the corresponding author on reasonable request.
